# Effective Formation of Well-Defined Polymeric Microfibers and Nanofibers with Exceptional Uniformity by Simple Mechanical Needle Spinning

**DOI:** 10.3390/polym10090980

**Published:** 2018-09-03

**Authors:** Hoik Lee, Yuma Inoue, Myungwoong Kim, Xuehong Ren, Ick Soo Kim

**Affiliations:** 1 Nano Fusion Technology Research Group, Division of Frontier Fibers, Institute for Fiber Engineering (IFES), Interdisciplinary Cluster for Cutting Edge Research (ICCER), Shinshu University, Nagano 386-8567, Japan; hoik0822@gmail.com (H.L.); 18fs304h@shinshu-u.ac.jp (Y.I.); 2 Department of Chemistry and Chemical Engineering, Inha University, Incheon 22212, Korea; mkim233@inha.ac.kr; 3 Key Laboratory of Eco-textiles of Ministry of Education, College of Textiles and Clothing, Jiangnan University, Wuxi 214122, China; xuehongr@hotmail.com

**Keywords:** nanofiber, fabrication, mechanical force, needle spinning, uniform nanofibers

## Abstract

The fabrication of nanofibers with a mechanical force has attracted increasing attention owing to its facile and easy fabrication. Herein, we demonstrate a novel and facile fabrication technique with the mechanical force, needle spinning, which utilizes a needle tip to draw a polymer solution to form fibrous structures. We studied the effect of the processing parameters to the nanofiber structure, namely, the pulling away speed, pulling away distances, needle size, and polymer concentration, which were systemically controlled. As the needle spinning provides an effective route to adjust those parameters, highly uniform nanofibers can be achieved. There are clear tendencies in the diameter; it was increased as the polymer concentration and needle size were increased, and was decreased as the pulling away distance and pulling away speed were increased. Needle spinning with a precise control of the processing parameter enables us to readily fabricate well-defined nanofibers, with controlled dimensions in diameter and length; plus, single nanofibers also can be easily formed. Those features cannot be realized in common spinning process such as electrospinning. Therefore, this technique will lead to further development of the use of mechanical force for nanofiber fabrication and will expand the range of nanofibers applications.

## 1. Introduction

Nanofibers have emerged as one of the promising one-dimensional nanomaterials, owing to their unique physicochemical properties, for example, their exceptionally large surface area to volume ratio and their long length with a small diameter in the nanometer scale [1,2]. Also, nanofibers can be easily engineered to introduce desired properties, for example, a high porosity with excellent pore interconnectivity [3]. Their unique characteristics make them attractive candidates for advanced applications, such as electronic devices [4,5], environmental remediation by filtration [6,7], and biomedical applications [8,9]. The nanofibers are fabricated by various techniques; a popular method is electrospinning. Electrospinning has been regarded as the most widely utilized way to produce nanofibers with a controlled fiber diameter on a large scale [10,11]. In this technique, a strong electrical force is utilized in order to draw a polymer solution into fine filaments. When a sufficiently high voltage is applied to the polymer solution at the tip of the syringe needle, highly charged droplets are ejected from the tip. The droplets are then stretched out to form a nanofiber as a result of the electrostatic repulsion force in the charged droplets, which counteracts the effect of the surface tension. The use of electrical force is beneficial for producing nanofibers on a large scale, however, it also results in several limitations in the nanofiber fabrication. Firstly, the solubility of polymers, in particular solvents, and their low electrical conductivity are known to limit the generalization of electrospinning in order to produce nanofibers [12]. Secondly, the use of the high electrical voltage leads to an excessive use of energy in the process, and therefore, the process can also require a high cost. Thirdly, the electrospun nanofibers are generally randomly placed on the substrate for collection, unless specific fabrication methods and setups are not employed. Thus, positioning the individual nanofibers at desired locations is difficult and, as a result, applying nanofibers into electronic device applications is highly limited [13]. Even the fabrication of single nanofibers is quite challenging as well. There are also many other techniques, such as melt blowing, wet spinning, and co-extrusion techniques. However, most techniques typically result in the fibers in the micrometer range, with low uniformity.

To address the challenges in electrospinning and the other techniques, there have been tremendous efforts to develop novel strategies to effectively fabricate nanofibers without the use of electrical force [14,15]. Mechanical force is a promising and beneficial alternative to the electrical force, when the nanofilament is drawn from the polymer solution. The use of mechanical force can avoid the high cost and excessive use of energy in production. More importantly, a much wider range of polymers and solvents can be applied to the process, because electrical conductivity is not a significant parameter [12]. Also, the orientation of the fibers can be easily controlled along the direction of the applied force, providing a degree of freedom to control the orientation, alignment, and position on a collection substrate. Recently, we reported a novel method for fabricating nanofibers using mechanical force, namely hand spinning, to address the challenges in electrospinning [16]. The hand spinning process was developed by mimicking a finger-pulling motion using highly viscous liquid glue, which forms a microfiber structure from the glue material. The simple pulling-out motion of two plates, where a certain amount of polymer solution is sandwiched in between, generates a mechanical stretching force on the polymer solution. As a result, filaments with a small diameter are formed during the stretching motion. Although this method successfully overcomes the limitations of electrospinning, it also suffered from the issue of nanofiber size control; the handspun nanofibers were not finely controllable and uniform in size (i.e., fiber diameter), because the polymer solution cannot be evenly supplied on the entire surface of the pulling plate. Moreover, the meniscus generated at the initial moment of the pulling-away motion is not uniform.

**Figure 1:**
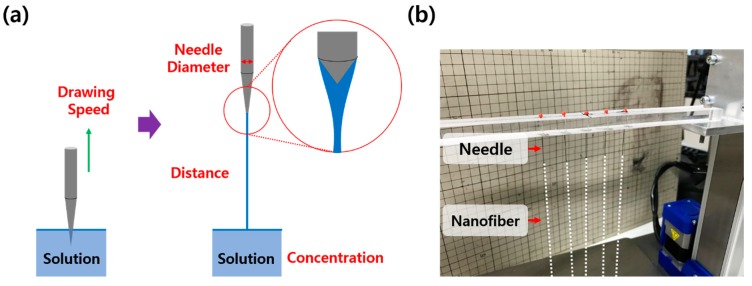
(**a**) Schematic illustration of a needle spinning apparatus and (**b**) a photograph of a needle spinning apparatus.

To achieve a controlled nanofiber size with a high uniformity, while keeping the benefits of hand spinning, we developed a method using needles instead of plates as a tool to draw the polymer solution with mechanical force, namely needle spinning. The schematic illustration of needle spinning and a photograph of a needle spinning apparatus are presented in Figure 1. By needle spinning, a single nanofiber can be readily fabricated by simply dipping a needle in the polymer solution, located at the bottom, and then the needle is drawn out of the solution. This setup makes the shape of the meniscus at the tip of the different needles extremely regular, which cannot be achieved with hand spinning at all. Consequently, highly uniform nanofibers in size and length can be fabricated. This technique could have a drawback regarding the throughput of needle spinning, as it produces a single fiber at once from single needle. However, the current instrument is for a proof of concept for fabricating the nanofiber with a precisely controlled size and high uniformity. The simplicity of this method should provide ready modifications for the design of the spinning setup, leading to an increase of the productivity. In our case, five different needles were equipped.

Herein, we demonstrate the fabrication of poly (ethylene oxide) nanofibers by needle spinning. The fabricated nanofibers exhibited a smooth surface and well-controlled diameter from ~1.4 to ~0.4 μm, by controlling the processing parameters, namely: the pulling away speed (*S*_PA_, mm/sec), pulling away distance (*d*_PA_, mm), needle size (*d*_N_, mm), and polymer concentration (*c*, wt. %). The size and length of all of the nanofibers were highly uniform, the standard deviation of the nanofiber diameter was typically under 10% of the average diameter, and the length of all of the nanofibers was determined by the pulling away distance. The length of the nanofiber was extended up to 0.5 m with our simple setup, strongly suggesting that an exceptionally high aspect ratio can be achieved. We further found that the diameter of the nanofiber can be systematically controlled by varying the process parameters mentioned above. The following clear tendencies were observed: the nanofiber diameter was increased as the polymer concentration (and viscosity) and needle size were increased, and the diameter was decreased when the pulling away distance and pulling away speed were increased. These studies provide an effective route to obtain well-defined nanofibers with desired dimensional parameters, and also elucidate the fundamental principles for a mechanically driven nanofiber formation process. Our method should be useful for fabricating well-defined single nanofiber or nanofibers, with a high uniformity compared to other conventional methods.

## 2. Experimental 

### 2.1. Preparation of Polymer Solution

A certain amount of poly (ethylene oxide) ([PEO] Mw ~8000 kg/mol, Sigma Aldrich, Co., St. Louis, MO, USA) was dissolved in deionized water. The concentration of the PEO aqueous solution was varied from 2 wt. % to 3 wt. %. Sodium dodecyl sulfate (SDS, <0.4 wt. %, Nacalai Tesque, Inc., Kyoto, Japan) was added to the prepared solution in order to lower the surface tension. The mixture was further homogenized by sonication for 3 h.

### 2.2. Nanofiber Fabrication via Needle Spinning

The experimental setup for needle spinning is shown in Figure S1. This homemade needle spinning apparatus consists of five needles on the top, a z-axial motor for the drawing needles, and a chamber on the bottom that contains a polymer solution. The apparatus was designed to control various spinning processing parameters, that is, the pulling away speed (*S*_PA_, mm/s), pulling away distance (*d*_PA_, mm), and the diameter of needle (*d*_N_, mm). The default values of the parameters *S*_PA_, *d*_PA_, *d*_N_, and the concentration of the polymer solution (*c*, wt. %) were set to 1000 mm/sec, 500 mm, 0.12 mm, and 2 wt. %, respectively. In order to examine the effect of each parameter on the nanofiber formation, we conducted the needle spinning with a variation of *S*_PA_ from 50 to 1000 mm/sec, and *d*_PA_ from 100 to 500 mm, *d*_N_ from 0.12 to 1.22 mm, and a polymer concentration from 2 wt. % to 3 wt. %. The resulting individual nanofibers were collected, and were then subjected to morphological studies using scanning electron microscopy (SEM, JSM-6010LA, JEOL, Tokyo, Japan). The size of the nanofiber was characterized by measuring the diameter of the nanofibers from the SEM micrographs, using image analysis software (ImageJ, version 1.49, National Institutes of Health, Bethesda, Rockville, MD, USA). The nanofibers were taken from the same operation on each of the five needles, and were repeated 10 times. The average diameter and standard variation values were calculated from 50 fibers, and the representative scanning electron microscopy (SEM) images have been presented.

## 3. Results and Discussion

**Figure 2:**
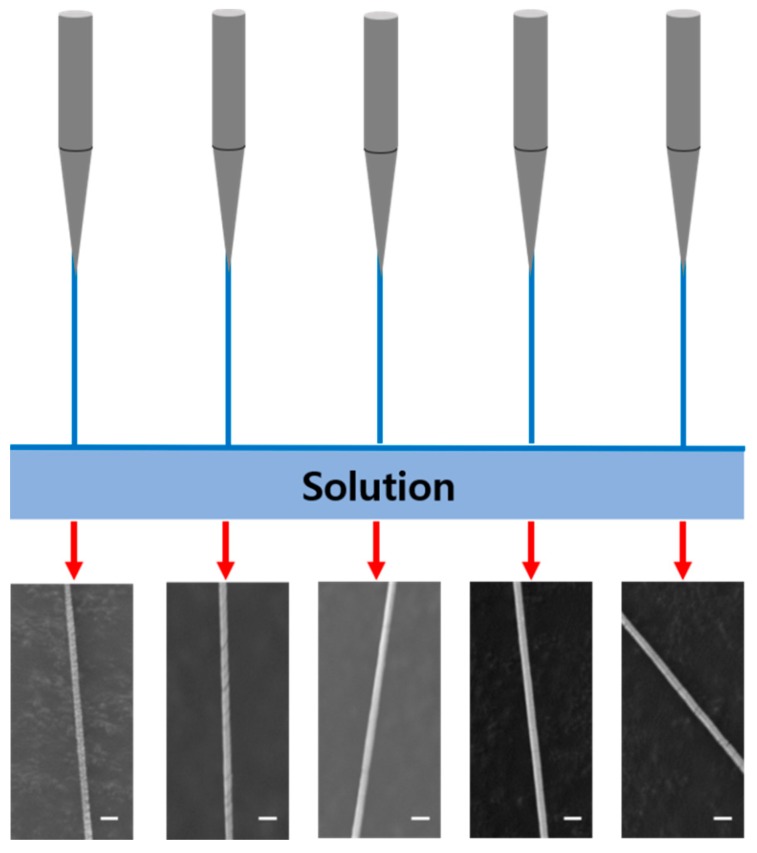
Schematic illustrations of the nanofiber fabrication process via needle spinning, and corresponding SEM images of a nanofiber fabricated from each needle (SEM scale bar = 2 μm).

The most important improvement of the needle spinning, when compared to other nanofiber fabrication techniques, is that it is capable of fabricating highly uniform nanofibers. With our homemade needle spinning apparatus, we aimed to precisely control all of the processing parameters, including the meniscus shape on the needle tip, which is a significant factor in the formation of a nanofiber. The morphology of five different individual nanofibers simultaneously formed by five needles is shown in in Figure 2. The resulting nanofibers exhibit the same morphology with almost same size. The diameters of the fabricated nanofibers extracted from the SEM images in Figure 2 are 493, 494, 499, 501, and 490 nm, respectively (from left to right). The average diameter of the nanofibers was 495.4 ± 4.5 nm, indicating that the size of the nanofibers is highly uniform compared to other nanofiber fabrication methods. The nanofibers formed with simple hand spinning typically have a much broader size distribution due to the unevenness of the meniscus formed during the spinning process. However, needle spinning enables achieving an identical meniscus on each needle when the spinning is carried out repeatedly, leading to an extremely higher uniformity.

**Figure 3:**
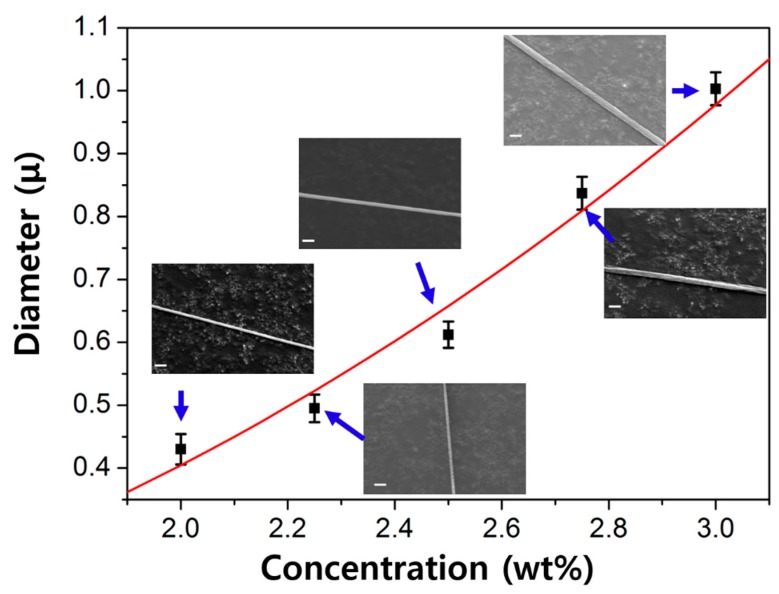
A plot of diameter as a function of polymer concentration, and corresponding SEM images at each concentration (SEM scale bar = 2 μm).

The needle spinning process can precisely control the varying processing parameters, that is, the concentration of the polymer solution, size of needle, pulling away distance, and pulling away speed. Firstly, we varied the concentration of the PEO aqueous solution from 2 wt. % to 3 wt. %. The concentration is known to be a significant factor in other spinning process [17]. In electrospinning, the applied voltage should overcome the surface tension of the solution so as to draw nanofiber filaments [18]. To pull out the solution drop with a high viscosity, a high enough force is necessary, which is applied by the strong electric field in the electrospinning process for successful nanofiber formation. Therefore, the increase of the concentration of the solution is limited in electrospinning, as it simultaneously increases the viscosity of the solution. On the contrary, as the needle spinning relies only on a simple mechanical force, the solution with a wide range of viscosity can be utilized for the nanofiber formation process. Figure 3 represents the correlation of the polymer solution concentration to the diameter of the needle-spun nanofiber (*D*) extracted from representative SEM images. As can be seen from the SEM images, all of the needle-spun nanofibers exhibited a bead-free morphology with a smooth surface. The plot was fitted with an exponential function of the concentration, *D* (nm) = *α·c^ν^* (*α* = 8.94 × 10^−2^; *ν* = 2.18). It is known that *D* of the electrospun nanofiber scales to the solution viscosity with the relationship of *D* ∝ *η^β^* [19]. As the viscosity of the aqueous PEO solutions increases when the solution becomes more concentrated [20], it implies that the size of the nanofibers fabricated by needle spinning is also closely related to viscosity, in a similar trend to electrospinning. Thus, controlling the concentration of the polymer solution can adjust the diameter of the needle-spun nanofiber.

**Figure 4:**
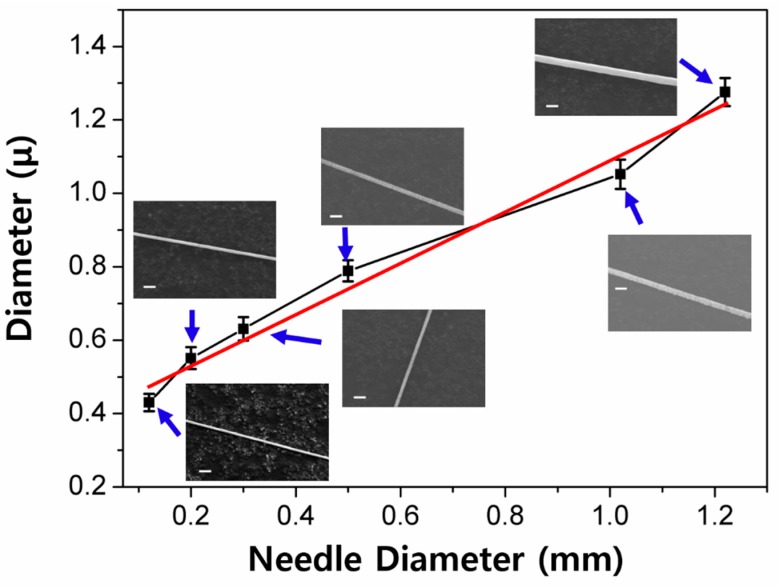
A plot of diameter as a function of needle diameter, and corresponding SEM images at each needle diameter (SEM scale bar = 2 μm). The red line represented a linear fitting.

Another processing parameter, the diameter of the needle (*d*_N_), was found to also significantly affect the diameter of the nanofiber. In the needle spinning, the size of the needle determines the shape of the drawn fiber filament on the tip, when the polymer solution is drawn by the tip. In the spinning process, firstly, the needle tip is slightly immersed in the polymer solution. Then, the needle is pulled out of the solution. Through the drawing of the needle, the polymer fiber filament is formed between the needle tip and the surface of the solution. The diameter of the filament formed at initial moment of needle spinning is likely equal to the size of the needle tip. Subsequently, pulling out the needle from the solution gradually reduces the fiber diameter. Hence, the size of the needle plays a critical role in the control of the nanofiber diameter. In order to examine this aspect, we varied the diameter of the needle tip to carry the needle spinning out. Figure 4 shows the variation of the fiber diameter as a function of the diameter of the needle (0.12–1.22 mm). The concentration, pulling away speed, and pulling away distance were fixed at 2.0 wt. %, 1000 mm/sec, and 500 mm, respectively. Under these processing conditions, all of the resulting nanofibers were well-defined with highly uniform and smooth morphologies. It was observed that the size of the formed nanofiber is systematically increased upon the increase of the needle size. It is worth noting that the nanofiber diameter scales linearly to the needle size. The linear fitting of the experimental data resulted in a simple empirical equation, *D* (μm) = 0.40 + 0.69 *d*_N_ (mm). These results strongly suggest that the size of the nanofiber can be precisely controlled by simply changing the needle size.

**Figure 5:**
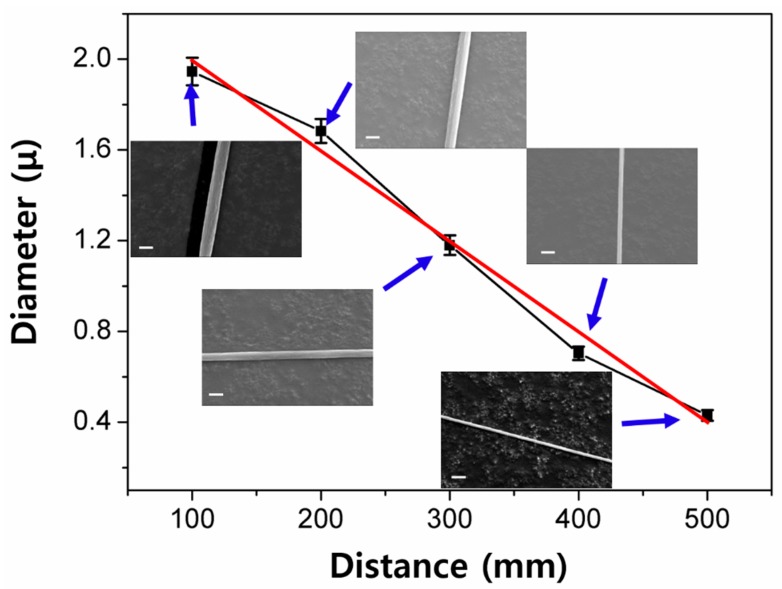
A plot of diameter as a function of distance, and corresponding SEM images at each distance (SEM scale bar = 2 μm). The red line represented a linear fitting.

As mentioned above, the fiber filament is initially formed between the needle tip and the surface of the polymer solution. This initially formed filament is elongated by drawing the needle along the z-axis. In this process, the diameter of the fiber becomes reduced to smaller size as it is pulled further out. To examine the effect of the pulling away distance variation, we fabricated the filaments with the variation of *d*_PA_ from 100 to 500 mm. The concentration, *d*_N_, and *S*_PA_ were fixed at 2 wt.%, 0.12 mm, and 1000 mm/sec, respectively. Under these conditions, the variation of *d*_PA_ did not affect the surface morphologies and the uniformity of nanofibers without any beads along the fibers, as shown in the SEM images of Figure 5. The diameter of the nanofiber decreases with the increase of *d*_PA_. The highest and lowest diameter values (and length) were found to be 1.94 ± 0.061 μm (100 mm) and 0.43 ± 0.024 μm (500 mm), respectively. A simple linear fitting of the relation between *D* and *d*_PA_ results in an empirical equation of *D* (μm) = 2.398 − 0.004·*d*_PN_ (mm). It should be noted that the pulling away distance largely affects the size of resulting fiber compared to the other parameters, that is, the concentration and needle size. The increase of the distance by five times, from 100 to 500 mm, led to a decrease of about four times in the diameter, from 1.94 to 0.43 μm. In cases of a variation of the concentration and needle size, the highest diameter can be found to be in the range of two to three times that of the lowest diameter value.

We note that the length of the nanofibers should be equal to *d*_PA_, indicating that a careful control of the processing conditions should allow for achieving the pre-selected diameter in the nanometer scale and length in the meter scale. The nanofibers with the diameter from the micro- to nano-meter range and the maximum fiber length of ~50 cm, can be achieved with current needle spinning setup. As the precise control of the fiber length was challenging in electrospinning, it is apparently a significant improvement.

**Figure 6:**
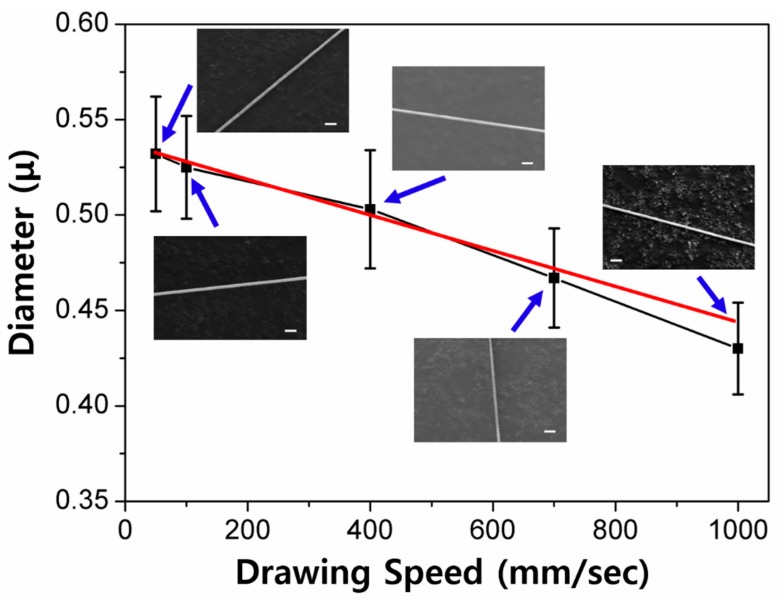
A plot of diameter as a function of the drawing speed, and corresponding SEM images at each drawing speed (SEM scale bar = 2 μm). The red line represented a linear fitting.

Figure 6 presents the relationship between the pulling away speed and the fiber diameter, which also shows the inverse linear correlation. The pulling away speed was varied from 50 to 1000 mm/sec, and the diameter of the fabricated fibers was controlled from ~0.532 ± 0.03 μm to 0.430 ± 0.024 μm. Interestingly, the diameter is clearly affected by the pulling away speed, although the size change is not dramatic compared to the other processing parameters. This phenomenon can be rationalized by two possible scenarios. Firstly, the fast drawing does not allow enough time for the polymer chains in the filament to diffuse during the needle spinning process. Inevitably, the polymer chains reside in a relatively small volume in the filament, leading to the formation of thinner nanofibers. Secondly, when the polymer solution is quickly pulled out by the needle tip, a strong stretching force is applied along the filament. This stretching force can give more chances for the polymer chains to be more arranged along the z-axis. It is worth noting that the difference of the diameter values of the fibers fabricated with an *S*_PA_ of 500 and 1000 mm/sec is about 100 nm. That is, the pulling away speed does not significantly affect the fiber size, compared to the other parameters. Similar to other processing parameters, the fiber diameter also linearly scales to the pulling away speed, and the empirical equation generated by a linear fitting analysis was *D* (μm) = 0.54 − 9.08 × 10^−5^ *S*_PA_ (mm/sec). As shown in the equation, the small slope indicates that the drawing speed is not critical for determining the diameter.

## 4. Conclusions

The simple but innovative method, needle spinning, offers an effective and facile route for fabricating highly uniform nanofibers with a smooth surface morphology and the desired dimensions in diameter and length. The method uses needles as a drawing tool to make the shape of the meniscus at the tip extremely regular, which cannot be realized in hand spinning. The effect of the processing parameters of needle spinning, that is, the pulling away speed, pulling away distance, needle size, and polymer concentration, was carefully investigated. They significantly affect the fiber structure; the nanofiber becomes thicker as the polymer concentration and needle size increase, and becomes thin as the pulling away speed and pulling away distance increase. Those relationships were depicted by four equations, which can be an important and useful tool for designing nanofibers and for achieving the desired property in nanofibers. Those clear tendencies enable us to achieve well-defined nanofibers in diameter and length, with exceptional uniformity. Moreover, this technique is highly advantageous for making a single nanofiber or nanofibers with a higher uniformity than other conventional methods. Plus, it keeps the benefits of hand spinning, for example, wide options for the polymer and the solvent selections, the ability to align the anisotropic particles in the nanofiber, a low cost, and excessive use of energy, by avoiding a high electrical voltage in the production of the nanofibers. We envision that these advantages of needle spinning will enable us to move forward to more advanced micro- and nano-structure fabrication technology.

## Supplementary Materials

The following are available online at http://www.mdpi.com/2073-4360/10/9/980/s1, Figure S1: A photograph of the experimental setup for needles pinning.

## References

[1] Lee H., Koo J.M., Sohn D., Kim I.-S., Im S.S. (2016). High thermal stability and high tensile strength terpolyester nanofibers containing biobased monomer: Fabrication and characterization. RSC Adv..

[2] Lee H., Yamaguchi K., Nagaishi T., Murai M., Kim M., Wei K., Zhang K.-Q., Kim I.S. (2017). Enhancement of mechanical properties of polymeric nanofibers by controlling crystallization behavior using a simple freezing/thawing process. RSC Adv..

[3] Lee H., Nishino M., Sohn D., Lee J.S., Kim I.S. (2018). Control of the morphology of cellulose acetate nanofibers via electrospinning. Cellulose.

[4] Wu Q., Xu Y., Yao Z., Liu A., Shi G. (2010). Supercapacitors based on flexible graphene/polyaniline nanofiber composite films. ACS Nano.

[5] Chen L., Chen L., Ai Q., Li D., Si P., Feng J., Zhang L., Li Y., Lou J., Ci L. (2018). Flexible all-solid-state supercapacitors based on freestanding, binder-free carbon nanofibers@polypyrrole@graphene film. Chem. Eng. J..

[6] Kim J., Chan H.S., Bae G.N., Jung J.H. (2017). Electrospun magnetic nanoparticle-decorated nanofiber filter and its applications to high-efficiency air filtration. Environ. Sci. Technol..

[7] Kampalanonwat P., Supaphol P. (2010). Preparation and adsorption behavior of aminated electrospun polyacrylonitrile nanofiber mats for heavy metal ion removal. ACS Appl. Mater. Interfaces.

[8] Wahab J.A., Kee J.Y., Hoik L., Seong-Geun O., Soo H.D., Zeeshan K., Joon C.H., Soo K.I. (2018). Antibacterial efficacy of poly (vinyl alcohol) composite nanofibers embedded with silver-anchored silica nanoparticles. J. Biomed. Mater. Res. B.

[9] Xu T., Yang H., Yang D., Yu Z.-Z. (2017). Polylactic acid nanofiber scaffold decorated with chitosan islandlike topography for bone tissue engineering. ACS Appl. Mater. Interfaces.

[10] Kim M.H., Lee W.J., Lee D.H., Ko S.W., Hwang T.I., Kim J.W., Park C.H., Kim C.S. (2018). Development of nanofiber reinforced double layered cabin air filter using novel upward mass production electrospinning set up. J. Nanosci. Nanotech..

[11] Moon S., Gil M., Lee K.J. (2017). Syringeless electrospinning toward versatile fabrication of nanofiber web. Sci. Rep..

[12] Kei W., Byoung-Suhk K., Yuji E., Ick-Soo K. (2011). Fabrication of uniaxially aligned poly (propylene) nanofibers via handspinning. Macromol. Mater. Eng..

[13] Huang S., Zhao C., Pan W., Cui Y., Wu H. (2015). Direct writing of half-meter long CNT based fiber for flexible electronics. Nano Lett..

[14] Hwee K.G., Hyoryung N., WooSeok C., Taechang A., Geunbae L. (2018). Electrospinning nanofiber on an insulating surface with a patterned functional electrolyte electrode. Adv. Mater. Interfaces.

[15] Tokarev A., Asheghali D., Griffiths I.M., Trotsenko O., Gruzd A., Lin X., Stone H.A., Minko S. (2015). Touch- and brush-spinning of nanofibers. Adv. Mater..

[16] Lee H., Watanabe K., Kim M., Gopiraman M., Song K.-H., Lee J.S., Kim I.S. (2016). Handspinning enabled highly concentrated carbon nanotubes with controlled orientation in nanofibers. Sci. Rep..

[17] Phan D.-N., Lee H., Choi D., Kang C.-Y., Im S., Kim I. (2018). Fabrication of two polyester nanofiber types containing the biobased monomer isosorbide: Poly (ethylene glycol 1,4-cyclohexane dimethylene isosorbide terephthalate) and poly (1,4-cyclohexane dimethylene isosorbide terephthalate). Nanomaterials.

[18] Szentivanyi A.L., Zernetsch H., Menzel H., Glasmacher B. (2011). A review of developments in electrospinning technology: New opportunities for the design of artificial tissue structures. Int. J. Artif. Organs.

[19] Petras D., Slobodian P., Pavlínek V., Sáha P., Kimmer D. (2011). The effect of pvac solution viscosity on diameter of pvac nanofibres prepared by technology of electrospinning. AIP Conf. Proc..

[20] Holyst R., Bielejewska A., Szymanski J., Wilk A., Patkowski A., Gapinski J., Zywocinski A., Kalwarczyk T., Kalwarczyk E., Tabaka M. (2009). Scaling form of viscosity at all length-scales in poly (ethylene glycol) solutions studied by fluorescence correlation spectroscopy and capillary electrophoresis. Phys. Chem. Chem. Phy..

