# Supplementary material for: Effective Formation of Well-Defined Polymeric Microfibers and Nanofibers with Exceptional Uniformity by Simple Mechanical Needle Spinning
Source: Polymers (Basel). 2018 Sep 3;10(9):980. doi: 10.3390/polym10090980 (PMC6403702; doi:10.3390/polym10090980)
Supplement: Supplementary file 1 [file polymers-10-00980-s001.pdf]

Article

Effective Formation of Well-Defined Polymeric Microfibers and Nanofibers with Exceptional Uniformity by Simple Mechanical Needle Spinning

Hoik Lee [1], Yuma Inoue [1], Myungwoong Kim [2], Xuehong Ren [3] and Ick Soo Kim [1,*]

[1] Nano Fusion Technology Research Group, Division of Frontier Fibers, Institute for Fiber Engineering (IFES), Interdisciplinary Cluster for Cutting Edge Research (ICCER), Shinshu University, Nagano 386-8567, Japan; hoik0822@gmail.com (H.L.); 18fs304h@shinshu-u.ac.jp (Y.I.)

[2] Department of Chemistry and Chemical Engineering, Inha University, Incheon 22212, Korea; mkim233@inha.ac.kr

[3] Key Laboratory of Eco-textiles of Ministry of Education, College of Textiles and Clothing, Jiangnan University, Wuxi 214122, China; xuehongr@hotmail.com

* Correspondence: kimicksoo.gr@gmail.com; Tel.: +81-268-21-5439; Fax: +81-268-21-5482

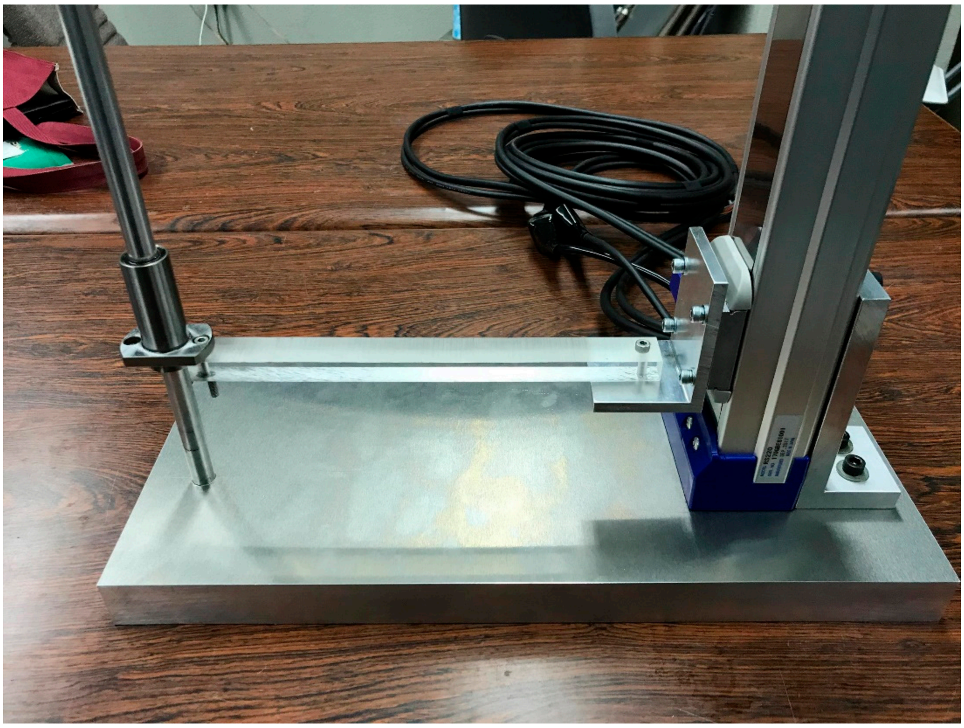

Figure S1. A photograph of the experimental setup for needlespinning.
